# Theoretical Study of As_2_O_3_ Adsorption Mechanisms on CaO surface

**DOI:** 10.3390/ma12040677

**Published:** 2019-02-25

**Authors:** Yaming Fan, Qiyu Weng, Yuqun Zhuo, Songtao Dong, Pengbo Hu, Duanle Li

**Affiliations:** 1 Research Institute of Petroleum Processing, SINOPEC, Beijing 100083, China; fanymthu@163.com (Y.F.); dongst.ripp@sinopec.com (S.D.); 2 Key Laboratory for Thermal Science and Power Engineering of the Ministry of Education, Department of Energy and Power Engineering, Tsinghua University, Beijing 100084, China; wqy17@mails.tsinghua.edu.cn (Q.W.); hupb18@mails.tsinghua.edu.cn (P.H.); liduanle@163.com (D.L.); 3 Tsinghua University-University of Waterloo Joint Research Center for Micro/Nano Energy and Environment Technology, Tsinghua University, Beijing 100084, China; 4 Beijing Engineering Research Center for Ecological Restoration and Carbon Fixation of Saline–alkaline and Desert Land, Tsinghua University, Beijing 100084, China

**Keywords:** CaO, As_2_O_3_, DFT, adsorption

## Abstract

Emission of hazardous trace elements, especially arsenic from fossil fuel combustion, have become a major concern. Under an oxidizing atmosphere, most of the arsenic converts to gaseous As_2_O_3_. CaO has been proven effective in capturing As_2_O_3_. In this study, the mechanisms of As_2_O_3_ adsorption on CaO surface under O_2_ atmosphere were investigated by density functional theory (DFT) calculation. Stable physisorption and chemisorption structures and related reaction paths are determined; arsenite (AsO_3_^3−^) is proven to be the form of adsorption products. Under the O_2_ atmosphere, the adsorption product is arsenate (AsO_4_^3−^), while tricalcium orthoarsenate (Ca_3_As_2_O_8_) and dicalcium pyroarsenate (Ca_2_As_2_O_7_) are formed according to different adsorption structures.

## 1. Introduction

Arsenic is a hazardous element existing in fossil fuels such as coal and petroleum [1]. According to the properties of arsenic and its compounds, it has been classified as volatile trace element by Clark and Sloss [2]. During combustion or chemical industry processes, gaseous arsenic is released into the environment. Excess amounts of arsenic can pollute water and soil. The exposure of arsenic to human may lead to hyperpigmentation, keratosis, skin and lung cancers with high possibility [3,4]. Arsenic compounds (including inorganic arsine) have been identified as hazardous air pollutants by the US government since 1990 [5]. The concentration of atmospheric arsenic in China is 51.0 ng/m^3^, which is much higher than the limit of NAAQS (6.0 ng/m^3^, GB 3095-2012) and the limit of WHO (6.6 ng/m^3^, WHO) [6].

Combustion of fossil fuels, especially coal, is one of the main sources for anthropogenic emission of atmospheric arsenic [7]. It was estimated that 335.5 tons of atmospheric arsenic were emitted from Chinese coal-fired plants in 2010 [8]. In 2011, the US Environmental Protection Agency issued the Mercury and Air Toxics Standards (US, MATS, updated in 2016). An arsenic emission limit of 3.0 × 10^−3^ lb/MWh (approximately 0.41 μg/m^3^) was set for coal-fired power plants [9]. In Chinese coal-fired power plants, the control of arsenic still remains scarce, but there are increasing interests in understanding its transformation in flue gas and developing emission reduction techniques.

Under an oxidizing atmosphere, gaseous As_2_O_3_ should be the main form of arsenic combustion products [10]. It has been proven that CaO could adsorb As_2_O_3_ in the coal-fired flue gas, and the dominating products were arsenate (AsO_4_^3−^) [11,12,13,14]. CaO component in fly ash leads to the enrichment of arsenic [15,16,17,18]. R.O. Sterling [11] found that CaO could effectively adsorb As_2_O_3_ at 600 °C and 1000 °C; the adsorption products were Ca_3_As_2_O_8_ when O_2_ existed. Jadhav [12] studied the adsorption products of As_2_O_3_ on a CaO surface under O_2_ atmosphere between 300 °C and 1000 °C. X-ray photoelectron spectroscopy (XPS) and X-ray Diffraction (XRD) reflected that, when temperature was lower than 600 °C, the adsorption product was Ca_3_As_2_O_8_; when temperature was between 700 °C and 900 °C, the adsorption products was Ca_2_As_2_O_7_; and when temperature was as high as 1000 °C, the adsorption product was Ca_3_As_2_O_8_. He also revealed that SO_2_ and HCl played a weak role in adsorption. Li [13,14] studied the influence of CO_2_ and SO_2_ on the capture of As_2_O_3_ by CaO. The existence of SO_2_ and CO_2_ did not change the form of arsenic in adsorption products. The previous study certified the strong adsorption of As_2_O_3_ on CaO surface and the important role O_2_ played in the reaction. However, the acute toxicity and low concentration of arsenic significantly limit the experimental research of As_2_O_3_ adsorption. The adsorption mechanisms still remain unclear, especially the composition of adsorption active sites and product structures.

Quantum chemistry calculation based on density functional theory (DFT) has become an effective method to simulate structures [19] and surface reaction of volatile trace elements [20]. For example, the adsorption of As^0^ on a CaO (001) surface has been effectively studied by Zhang [21]. In this study, the adsorption structures and the detailed adsorption steps between the CaO surface and As_2_O_3_ (under O_2_ atmosphere) have been studied by advanced DFT calculation, with the aim to offer microscopic information about critical reactions, and thus, to provide guidance to develop more efficient adsorbents and related control technologies.

## 2. Methods and Modeling

### 2.1. Methods

The material studio CASTEP [22,23] module was applied in the DFT calculation. The GGA (Generalized Gradient Approximation) and PBE [24] (Perdew-Burke-Ernzerhof) were chosen to describe the exchange and correlation interactions. The electronic wave functions were expanded on a plane wave basis with cut-off energy of 380 eV. The ultra-soft pseudo potential was referred to describe the interactions between electrons and the ionic cores [25]. ‘The spin-polarized’ option was selected for ‘spin-unrestricted’ calculations [26]. The BFGS (Broyden-Flechter-Goldfarb-Shanno) optimization algorithm was chosen for geometry optimization [27]. The transition state and reaction path (intermediate states) was determined by using the complete Linear Synchronous Transit/Quadratic Synchronous Transit (LST/QST) method [28] and confirmed by the Nudged-Elastic Band (NEB) method [29].

The convergence criteria of geometry optimization included: (a) self-consistent field (SCF) of 5.0 × 10^−7^ eV/atom; (b) energy of 5 × 10^−6^ eV/atom; (c) displacement of 5 × 10^−4^ Å; (d) force of 0.01 eV/Å; and (e) stress of 0.02 GPa. The convergence of complete LST/QST method (RMS, Root Mean Square) was set to 0.05 eV/Å. The convergence criteria of NEB included: (a) energy of 1.0 × 10^−5^ eV/atom; (b) max force of 0.05 eV/Å; and (c) max displacement of 0.004 Å.

The adsorption energy (*E_ads_*) was defined as follows:

*E_ads_* = *E_pro_* − (*E_slab_* + *E_adsorbate_*) (1)

where *E_pro_* was the total energy of adsorption product, *E_slab_* was the total energy of the slab model, and *E_adsorbate_* was the total energy of isolated adsorbate As_2_O_3_ or O_2_ at its equilibrium geometry. A negative *E_ads_* value represented a stable adsorption system.

### 2.2. Modeling

The energy of CaO crystal cell was converged with 6 × 6 × 6 *k* points in the Monhorst-pack grid [30]. The equilibrium geometry of As_2_O_3_ and O_2_ was examined in a cell of 20 × 20 × 20 Å^3^ periodic box. As shown in Table 1, the values of the calculated bond lengths, angles, and lattice parameters are consistent with the data reported from the previous study, indicating the reliability of the calculation.

In our previous study, the CaO(001) slab model has been widely used for CO_2_ [35], Se^0^ [36] and SeO_2_ [37] heterogeneous adsorption reaction, in which the good consistency with experimental work has been proven. Similarly, a 4-layer 3 × 3-surface CaO (001) slab was modeled to describe the CaO surface between CaO and As_2_O_3_ in this study. The superficial two layers of atoms were relaxed while the rest layers were fixed [38]. The vacuum region between slabs was set to 10 Å to avoid interactions among periodic images [39]. The energy of slab models and related adsorption structures were converged with 2 × 2 × 1 *k* points in the Monhorst-pack grid. The detailed modeling process was put in the Supplementary Materials (Optimization of slab model section: Tables S1 and S2).

## 3. Results and Discussions

According to the spatial position of As_2_O_3_ and surface atoms distribution, three groups, including twenty-one possible As_2_O_3_ structures, were first modeled as the initial structures for optimization (provided in Figure S1). After the geometric optimization of the initial structures, plenty of adsorption structures were validated, then the possible reaction paths were calculated. Based on the minimal point of the reaction paths, additional stable structures were acquired. Most of the physisorption structures were similar in terms of structural pattern and close in terms of energy level; thus, three representative physisorption structures (adsorption energy higher than −100 kJ/mol [40]) were determined. Additionally, ten chemisorption structures (adsorption energy lower than −100 kJ/mol [40]) were identified. Based on these structures, various adsorption paths were finally confirmed. For briefness, the n^th^ physisorption structure was abbreviated as P_n_, while the n^th^ chemisorption structure was abbreviated as C_n_.

### 3.1. Stable Sorption Structures

#### 3.1.1. Stable Physisorption Structures

Three representative physisorption structures have been shown in Table 2. The dominating differences are the number of As_2_O_3_’s O bonded with superficial Ca and the distribution of the superficial Ca occupied by As_2_O_3_’s O. Three types of physisorption follow the crystal orientation <100>, <110> and <110>, respectively. Two or three superficial Ca is close to As_2_O_3_’s O, and the bond length is about 2.380 Å to 2.876 Å. The corresponding adsorption energy ranges from −65.8 kJ/mol to −58.4 kJ/mol. Based on electron density cloud, physisorption active sites are composed of superficial Ca atoms that interact with O of As_2_O_3_.

#### 3.1.2. Stable Chemisorption Structures

Ten chemisorption structures were obtained, with *E_ads_* ranging from −198.5 kJ/mol to −391.4 kJ/mol, which implies strong chemisorption. Superficial Ca is close to As_2_O_3_’s O, the bond length is about 2.269 Å to 2.528 Å, while superficial O is close to As_2_O_3_’s O, the bond length is 1.788 Å to 2.086 Å. According to electron density cloud and bong length, chemisorption active sites are superficial O atoms that interact with As of As_2_O_3_. According to the adsorption energy and structure (i.e., the positions of As and O), four categories were classified in Table 3:

Category I: As_2_O_3_’s As is located on the hollow site
Category II: All of As_2_O_3_’s O is located on or close to superficial Ca top site
Category III: As_2_O_3_ transforms into a spoon-shaped structure
Category IV: All of As_2_O_3_’s As is located on two neighboring superficial O top site

### 3.2. Adsorption Process

Due to the continuity of energy, the adsorption process can be characterized as an energy-drop process, including both physisorption and chemisorption.

#### 3.2.1. Transformation Process of Physisorption Structures to Chemisorption Structures

In the following part, the transition state number n is abbreviated as TS_n_, and the intermediate position number n is abbreviated as IP_n_, for short.

**Figure 1:**
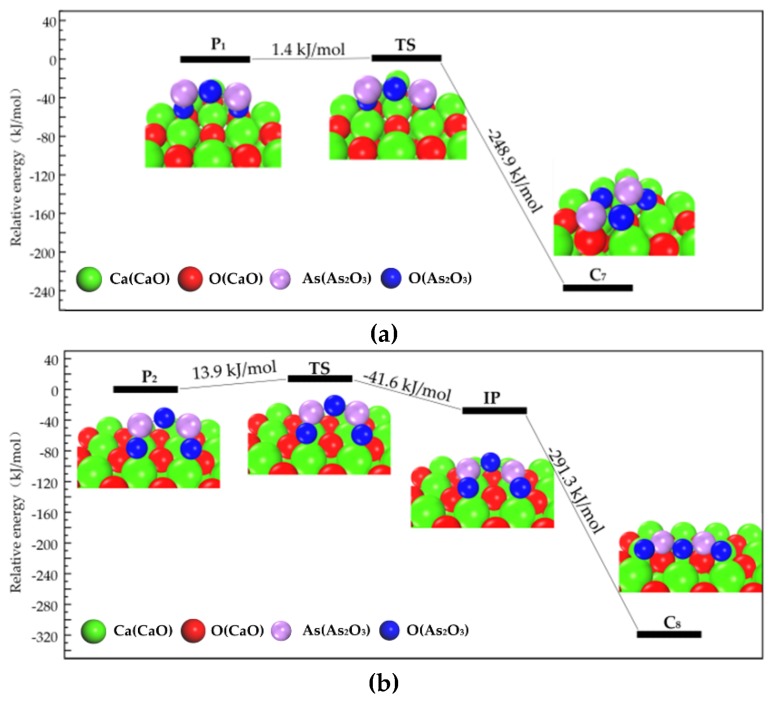

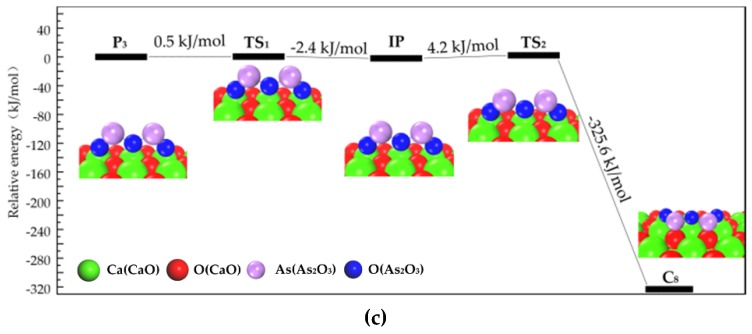
Structures and energies during transformation process of physisorption structures to chemisorption structures. (**a**) Reaction path of physisorption structure 1; (**b**) Reaction path of physisorption structure 2; (**c**) Reaction path of physisorption structure 3.

As shown in Figure 1, when As_2_O_3_ approaches the surface with vibration along the surface, the physisorption structure transforms into a chemisorption structure during one or two transition state. For instance, P_1_ to C_7_ (Figure 1a), P_2_ to C_8_ (Figure 1b) and P_3_ to C_8_ (Figure 1c). The energy barrier is low, from 1.4 kJ/mol to 13.9 kJ/mol, suggesting that the physisorbed As_2_O_3_ is not stable enough and could be easily transformed into chemisorption structures by thermal vibration.

#### 3.2.2. Transformation Process of Chemisorption Structures

Chemisorbed As_2_O_3_ gradually transforms into more stable structures. Different possible reaction paths were calculated. The four categories of chemisorption structures can be sorted by the *E_ads_* of each as Category IV < Category III ≈ Category II < Category I.

Category I has relatively high energy, i.e., relatively low stability, its transformation to Category II, III and IV could be triggered by molecular thermal vibration.

**Figure 2:**
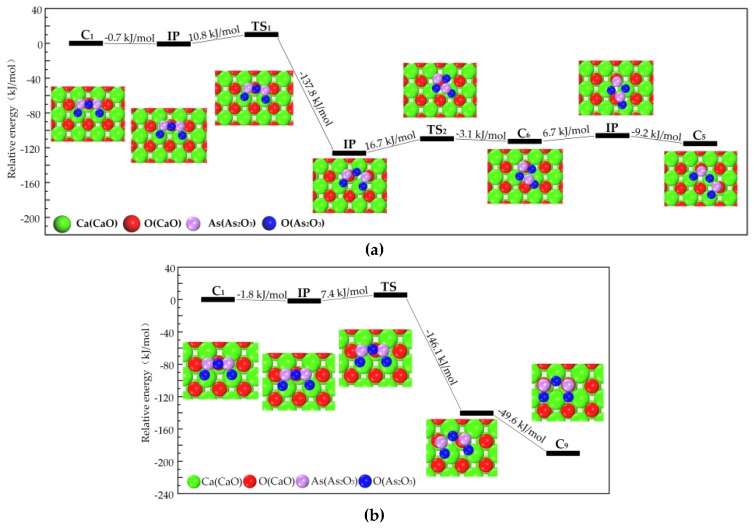
Transformation path of Category I. (**a**) Category I to Category II and III; (**b**) Category I to Category IV.

The pathway that Category I transforms to Category II is shown in Figure 2. Firstly, C_1_ transforms into C_6_ (Category II) and then C_5_ (Category III), with the energy barrier of 10.8 kJ/mol, 16.7 kJ/mol, and 6.7 kJ/mol, respectively. As shown in Figure 2, Category I transforms into Category IV along with another reaction path, the related energy barrier is 7.4 kJ/mol. The relatively low energy barrier suggests that Category I is not stable enough, and could easily transform to Category II, III and IV.

**Figure 3:**
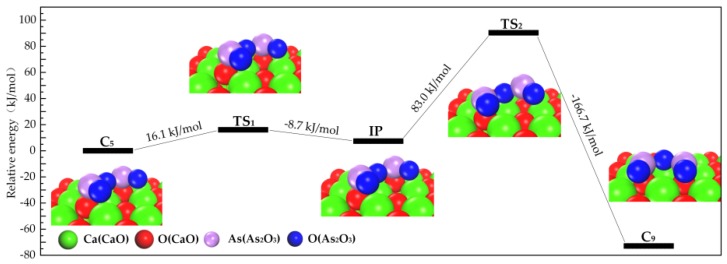
Transformation path of Category II.

The reaction path of Category II is shown in Figure 3. C_3_ firstly transforms into intermediate and then converts to C_9_. The corresponding energy barrier is 16.1 kJ/mol and 83.0 kJ/mol, proving that Category II transforms to Category IV with the special direction of thermal vibration.

**Figure 4:**
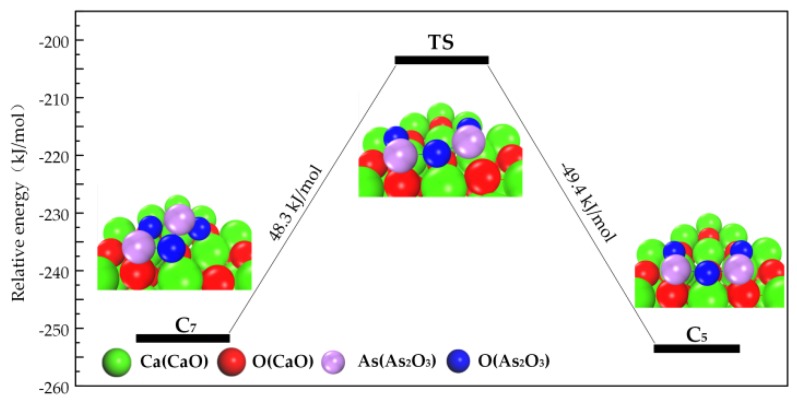
Transformation path of Category III.

Structures of Category III can transform into Category II, as shown in Figure 4. The spoon-shaped structure of As_2_O_3_ in C_7_ disappears and then overcomes a 48.3 kJ/mol energy barrier to transform to C_5_, indicating the conversion of Category III to Category II.

**Figure 5:**
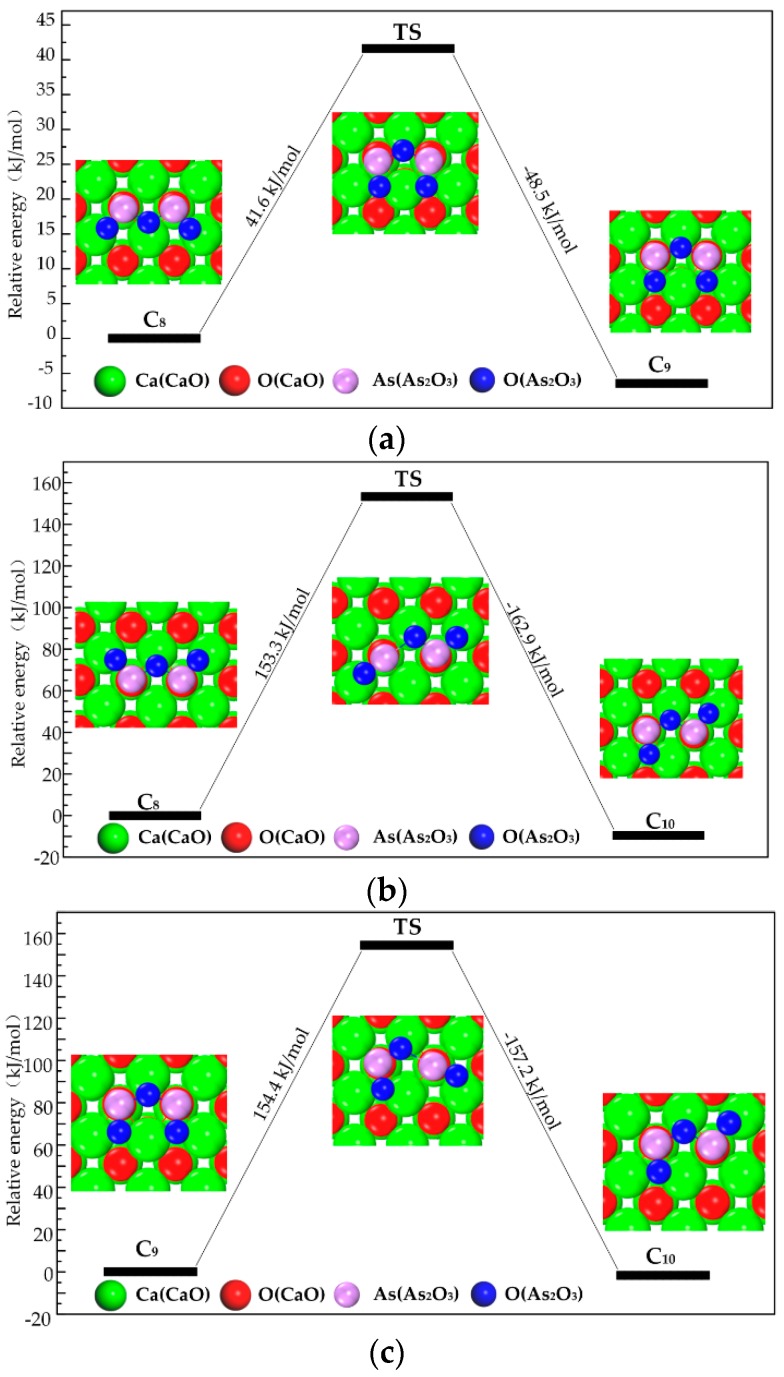
Transformation path of Category IV. (**a**) Reaction path of C_8_ to C_9_; (**b**) Reaction path of C_8_ to C_10_; (**c**) Reaction path of C_9_ to C_10_.

Category IV is the most stable category. C_8_, C_9_, and C_10_ can transform into each other (shown in Figure 5). As_2_O_3_’s As does not move during the transformation. When all of As_2_O_3_’s O in C_8_ vibrate, C_8_ converts to C_9_, and the energy barrier is 41.6 kJ/mol (Figure 5a). When one of As_2_O_3_’s O in C_8_ vibrates, C_8_ converts to C_10_, and the energy barrier is 153.3 kJ/mol (Figure 5b). When one of the oxygen atoms of As_2_O_3_ in C_9_ vibrates, C_9_ converts to C_10_, and the energy barrier is 154.4 kJ/mol (Figure 5c). The difference in energy barrier is caused by the movement distance of As_2_O_3_’s O being motivated by thermal vibration. In the first reaction, the movement distance of As_2_O_3_’s O is shorter than that in the second and third reactions, which demands relatively lower energy to overcome the energy barrier.

### 3.3. Path of the Reaction

According to above-mentioned processes, the reaction paths can be concluded as follows; firstly, the isolated As_2_O_3_ is physisorbed on a CaO surface (As_2_O_3_’s O weakly interacts with superficial Ca); secondly, the physisorbed As_2_O_3_ transforms to chemisorbed As_2_O_3_. (As_2_O_3_’s As interacts with superficial O); and thirdly, due to thermal vibration, the chemisorbed As_2_O_3_ transforms into more stable chemisorbed As_2_O_3_ (the position of As_2_O_3_’s O changed).

**Figure 6:**
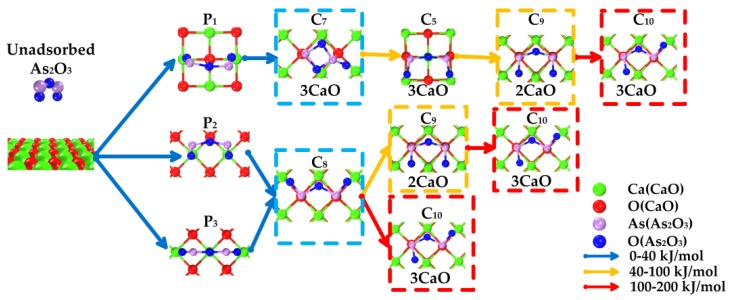
Overall adsorption paths of As_2_O_3_ on CaO.

The adsorption path of As_2_O_3_ was summarized as the process shown in Figure 6. These reactions could be classified as three types according to the energy barrier with the aim to reflect the intensity of the required reaction temperature. The number of superficial CaO occupied by As_2_O_3_ is also considered in order to describe the adsorption reaction equation.

Blue arrow: energy barrier is in the range of 0–40 kJ/mol, suggesting that reaction is likely to occur under a relatively low-temperature condition.

Yellow arrow: energy barrier is in the range of 40–100 kJ/mol, suggesting that reaction is likely to occur under a relatively medium-temperature condition.

Red arrow: energy barrier is in the range of 100–200 kJ/mol, suggesting that reaction is likely to occur under a relatively high-temperature condition.

Figure 6 reveals that three main reaction paths may exist:

1. As_2_O_3_ → P_1_ → C_7_ → C_9_ →C_10_;
2. As_2_O_3_ → P_2_ or P_3_ → C_8_ → C_9_ → C_10_;
3. As_2_O_3_ → P_2_ or P_3_ → C_8_ → C_10_.

Under a relatively low-temperature condition (blue arrow, 0–40 kJ/mol), the main products are C_7_ and C_8_ (blue grid). Three superficial Ca and one or two superficial O are involved in the reaction, representing three CaO participates in the adsorption. The adsorption equation could be written as:

As_2_O_3_ + 3 CaO → Ca_3_As_2_O_6_ (2)

Under a relatively medium-temperature condition (yellow arrow, 40–100 kJ/mol), the main products are C_9_. Two superficial Ca and two superficial O participate in the structure. The adsorption equation could be written as:

As_2_O_3_ + 2 CaO → Ca_2_As_2_O_5_ (3)

Under a relatively high-temperature condition (red arrow, 100–200 kJ/mol), the main product is C_10_. Three superficial Ca and two superficial O are involved in the reaction (hollow Ca represents 1/2 Ca atom). The adsorption equation could be written as:

As_2_O_3_ + 3 CaO → Ca_3_As_2_O_6_ (4)

With the reaction temperature increases, adsorption product changes from Ca_3_As_2_O_6_ to Ca_2_As_2_O_5_ and back to Ca_3_As_2_O_6_ again. Different microcosmic adsorption structures lead to different macroscopic products and reaction equation.

Besides, as shown in Figure S2, the paths of C_1_ transforming to other structures have been also been found. However, no possible paths which isolated or physisorbed As_2_O_3_ transforms to C_1_ has been found, implying C_1_ is unstable or nonexistent.

### 3.4. Partial Density of States (PDOS)

The change PDOS of As_2_O_3_ and CaO was put in the Supplementary Materials (Figure S3). For As_2_O_3_, the PDOS of physisorption structure 1, 2, and 3 are similar to each other. As the physisorption structure transforms to C_7_, the *p* state orbitals near Fermi level (from −0.6 eV to 1.9 eV) drift to lower energy level, meanwhile get energy splitting and orbital reorganization, caused by the changing of As_2_O_3_ structure and the combination between As_2_O_3_’s As and superficial O. When C_7_ transforms to C_5_, *s* state orbital (−17.2 eV) energy level splits into two peaks of −18.0 eV and −16.9 eV, which is caused by the As-O bond breaking and the bonding between As and superficial O. When C_5_ transform to C_8_, the *p* state orbital (3.7 eV) and *s* state orbital (−17.9 eV) energy level both split slightly. This is the result of the slight change in the surface distribution of As_2_O_3_. As the adsorption products have close energies and structures, PDOS of C_9_ and C_10_ are basically similar to C_8_.

For CaO slab surface, when an As_2_O_3_ molecule is physisorbed on the surface, little change of PDOS is detected. When As_2_O_3_ is chemisorbed, it can be seen that the superficial *p* orbitals around Fermi level (from −2.7 eV to 0.4 eV) drift to a lower energy range (from −5.8 eV to 0.2 eV). Moreover, a small peak (−16.8 eV) is separated from *s* orbitals (peak at −14.6 eV), proving that *s* orbitals participate in the chemisorption to some extent. Superficial *p* state orbitals near Fermi level play an important role in the chemisorption of As_2_O_3_. It suggests that the CaO surface’s property of capturing As_2_O_3_ might be improved by increasing the quantities of superficial *p* orbitals near Fermi level.

### 3.5. Influence of O_2_ on Adsorbed As_2_O_3_

Under the flue gas atmosphere, especially O_2_-containing atmosphere, O_2_ reacts with chemisorbed As_2_O_3_; i.e., arsenite (AsO_3_^3−^) is oxidized to arsenate (AsO_4_^3−^). As an example, two stable chemisorption structures (C_5_, C_9_) identified previously were presented in Table 4. The distance between As_2_O_3_’s As and O_2_’s O is 1.763–1.764 Å, which is close to the As-O bond length of As_2_O_3_ (1.628 Å). The distance between O_2_’s O and superficial Ca is 2.247–2.263 Å. According to the electron density cloud, one of O_2_’s O overlaps with As_2_O_3_’s As. The other O of O_2_ overlaps slightly with the superficial Ca.

Based on Figure 6 and Table 4, the reaction equation of adsorption under O_2_ atmosphere can be written as Equations (5)–(7), corresponding to low-temperature, medium-temperature, and high-temperature adsorption, respectively.

3CaO + As_2_O_3_ + O_2_ → Ca_3_As_2_O_8_ (5)

2CaO + As_2_O_3_ + O_2_ → Ca_2_As_2_O_7_ (6)

3CaO + As_2_O_3_ + O_2_ → Ca_3_As_2_O_8_ (7)

With the increase of reaction temperature, adsorption product changed from Ca_3_As_2_O_8_ to Ca_2_As_2_O_7_ and then to Ca_3_As_2_O_8_ in an O_2_-containing atmosphere. According to this research, the product under low-temperature and high-temperature conditions is Ca_3_As_2_O_8_ with different structures, i.e., crystalline form. Under a medium-temperature condition, the main product is Ca_3_As_2_O_7_.

Previous experimental research consistently reflected that the adsorption product with O_2_ existence is AsO_4_^3−^, while different opinions existed regarding the adsorption structures. The study of Jadhav [12] found that the adsorption product obtained under 500 °C was mainly Ca_3_As_2_O_8_ (JCPDS No.01-0933). Under 700 °C and 900 °C, the product was Ca_2_As_2_O_7_ (JCPDS No.17-0444). When the temperature increased to 1000 °C, the reaction product was Ca_3_As_2_O_8_ (JCPDS No.26-0295). Mahuli [41] (600 °C and 1000 °C) and Sterling [11] (800 °C) found that the adsorption product was Ca_3_As_2_O_8_ (JCPDS No. 26-0295), while the sorbent used by Mahuli was Ca(OH)_2_. Li [13] found that the product obtained under 600 °C mainly belonged to Ca_3_As_2_O_8_ crystal structure (JCPDS No. 01-0933), and another kind of Ca_3_As_2_O_8_ crystal (JCPDS No. 73-1928) was identified for the products obtained under 800 °C and 1000 °C.

The role of temperature on adsorption product transformation is qualitatively described. The more detailed description of the product layer development is associated with many other factors, such as the concentration and flow rate of As_2_O_3_ and O_2_, and the quantity and granular size of CaO. The quantitative description of the adsorption process is still a very difficult challenge. Nevertheless, the DFT calculation findings revealed by this study could directly explain the experimental results obtained by previous researchers, which might provide some meaningful insight to understand the process of As_2_O_3_ adsorption on CaO.

## 4. Conclusions

The mechanisms of As_2_O_3_ adsorption on a CaO surface have been studied by using DFT calculation; conclusions are as follows:

(1) Physisorption active sites are composed of superficial Ca atoms that interact with O of As_2_O_3_. Chemisorption active sites are superficial O atoms that interact with As of As_2_O_3_;
(2) The adsorption process can be described as follows: the isolated As_2_O_3_ molecule is firstly adsorbed on the CaO surface by physisorption, and then physisorbed As_2_O_3_ will transform to chemisorbed As_2_O_3_. Due to thermal vibration, the chemisorbed As_2_O_3_ would overcome the energy barrier and transform to a more stable chemisorbed As_2_O_3_ state. The adsorption product is AsO_3_^3−^;
(3) The adsorption products of As_2_O_3_ under an O_2_-containing atmosphere are AsO_4_^3−^. The adsorption product’s structure is influenced by the adsorption temperature. Under relatively low-temperature, the product is Ca_3_As_2_O_8_; under relatively medium-temperature, the product is Ca_3_As_2_O_7_; and under relatively high-temperature, the product is Ca_3_As_2_O_8_.

The consistency between DFT calculation and the previous experiments proves high possibilities to design and optimized the CaO-based adsorbents by modifying O sites or other elements. Besides, other flue gases such as SO_2_ or CO_2_ can be involved in the following study to achieve materials design under real flue gas conditions. The optimized CaO-based adsorbents should be of high industrial value, could be applied in the injection of limestone into the furnace, CaO looping reactor, and dry desulfurization, etc.

## Figures and Tables

**Table 1:** Calculated lattice parameters, bond lengths, and bond angles.

Substance	Previous Data	Simulated Data
CaO [31,32]	4.836 Å/4.807 Å	4.837 Å
As_2_O_3_ [33]	As–O bond 1.794 Å	As–O bond 1.814 Å
	As–O bond 1.610 Å	As–O bond 1.622 Å
	O–As–O angle 106.3°	O–As–O angle 111.2°
	As–O–As angle 133.8°	As–O–As angle 141.8°
O_2_ [34]	O–O 1.210 Å	O–O 1.240 Å

**Table 2:** Stable physisorption structures, adsorption energy, electron density cloud, and *E_ads_*.

Name	Top View	Front View	Electron Density Cloud	Structure Details	*E_ads_*
P_1_	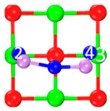	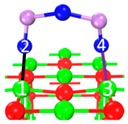	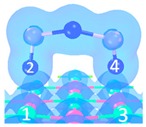	Bond_12_: 2.450 Å Bond_34_: 2.469 Å	−65.8 kJ/mol
P_2_	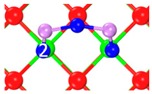	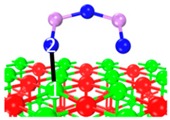	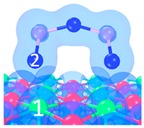	Bond_12_: 2.380 Å	−62.6 kJ/mol
P_3_	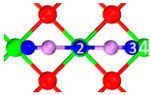	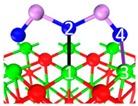	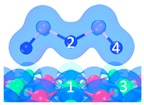	Bond_12_: 2.876 Å Bond_34_: 2.539 Å	−58.4 kJ/mol
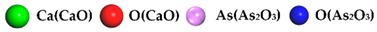					

**Table 3:** Chemisorption structures, adsorption energy, electron density cloud and *E_ads_*.

Category	Name	Top View	Front View	Electron Density Cloud	Structure Details	*E_ads_*
I	C_1_	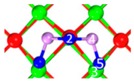	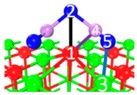	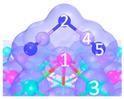	Bond_12_: 2.635 Å Bond_14_: 2.086 Å Bond_35_: 2.360 Å	−198.5 kJ/mol
II	C_2_	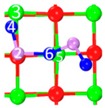	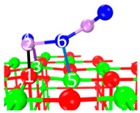	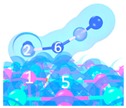	Bond_12_: 1.858 Å Bond_34_: 2.360 Å Bond_56_: 2.386 Å	−222.1 kJ/mol
II	C_3_	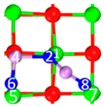	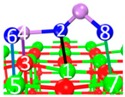	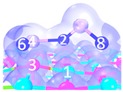	Bond_12_: 2.424 Å Bond_34_: 1.815 Å Bond_56_: 2.314 Å Bond_78_: 2.490 Å	−274.4 kJ/mol
II	C_4_	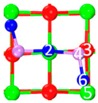	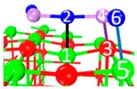	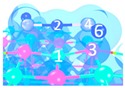	Bond_12_: 2.269 Å Bond_34_: 1.949 Å Bond_56_: 2.391 Å	−292.0 kJ/mol
II	C_5_	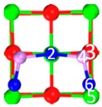	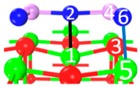	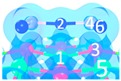	Bond_12_: 2.293 Å Bond_34_: 1.943 Å Bond_56_: 2.298 Å	−315.1 kJ/mol
III	C_6_	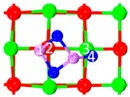	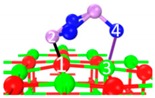	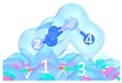	Bond_12_: 1.815 Å Bond_34_: 2.355 Å	−302.3 kJ/mol
III	C_7_	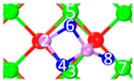	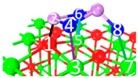	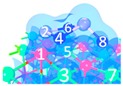	Bond_12_: 1.788 Å Bond_34_: 2.528 Å Bond_56_: 2.514 Å Bond_78_: 2.422 Å	−314.0 kJ/mol
IV	C_8_	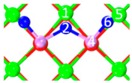	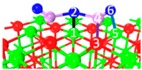	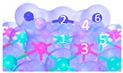	Bond_12_: 2.357 Å Bond_34_: 1.901 Å Bond_56_: 2.298 Å	−381.7 kJ/mol
IV	C_9_	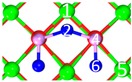	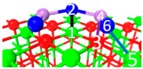	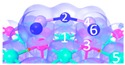	Bond_12_: 2.472 Å Bond_34_: 1.869 Å Bond_56_: 2.503 Å	−388.6 kJ/mol
IV	C_10_	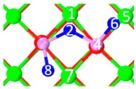	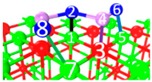	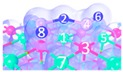	Bond_12_: 2.382 Å Bond_34_: 1.894 Å Bond_56_: 2.299 Å Bond_78_: 2.392 Å	−391.4 kJ/mol
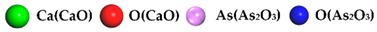						

**Table 4:** Stable chemisorption structures under O_2_ atmosphere, adsorption energy, electron density cloud and *E_ads_*.

Name	Top View	Front View	Electron Density Cloud	Structure Details	*E_ads_*
C_5_ under O_2_	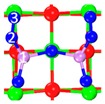	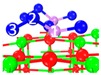	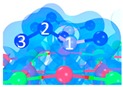	Bond_12_: 1.764 Å Bond_34_: 1.452 Å Bond_56_: 2.263 Å	−165.2 kJ/mol
C_8_ under O_2_	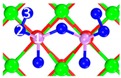	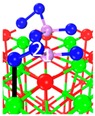	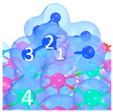	Bond_12_: 1.763 Å Bond_34_: 1.599 Å Bond_56_: 2.427 Å	−174.4 kJ/mol
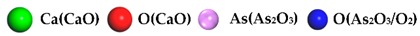					

## Supplementary Materials

The following are available online at https://www.mdpi.com/1996-1944/12/4/677/s1. Table S1: Changes in physical and chemical properties of different surface size; Table S2: Changes in physical and chemical properties of different layers; Figure S1: Initial adsorbate structures; Figure S2: Paths and structures of the physisorption and chemisorption reaction from chemisorption structure 1; Figure S3: PDOS of As_2_O_3_ and CaO surface during physisorption and chemisorption (a. PDOS of As_2_O_3_ molecule; and b. PDOS of CaO surface).
