# Supplementary material for: Theoretical Study of As2O3 Adsorption Mechanisms on CaO surface
Source: Materials (Basel). 2019 Feb 25;12(4):677. doi: 10.3390/ma12040677 (PMC6416740; doi:10.3390/ma12040677)
Supplement: Supplementary file 1 [file materials-12-00677-s001.pdf]

materials

Supplementary Materials

Theoretical Study of As_2O_3 Adsorption Mechanisms on CaO surface

Yaming Fan [1,2,†], Qiyu Weng [2,3,4,†], Yuqun Zhuo [2,3,4,*], Songtao Dong [1], Pengbo Hu [2,3,4] and Duanle Li [2,3,4]

1. Research Institute of Petroleum Processing, SINOPEC, Beijing 100083, China; fanymthu@163.com (Y.F.); dongst.ripp@sinopec.com (S.D.)
2. Key Laboratory for Thermal Science and Power Engineering of the Ministry of Education, Department of Energy and Power Engineering, Tsinghua University, Beijing 100084, China; wqy17@mails.tsinghua.edu.cn (Q.W.); hupb18@mails.tsinghua.edu.cn (P.H.); liduanle@163.com (D.L.)
3. Tsinghua University-University of Waterloo Joint Research Center for Micro/Nano Energy and Environment Technology, Tsinghua University, Beijing 100084, China
4. Beijing Engineering Research Center for Ecological Restoration and Carbon Fixation of Saline–alkaline and Desert Land, Tsinghua University, Beijing 100084, China

* Correspondence: zhuoyq@mail.tsinghua.edu.cn

† These authors contributed equally to this work

Optimization of slab model

CaO (001) is a typical surface in describing CaO [1,2] surface. The surface size (1×1, 2×2, 3×3, 4×4) and layers (2, 3, 4, 5 and 6) of the slab models were optimized to their physical and chemical properties. The physical property included the z_{layers} (layer thickness) and $z_{Ca\text{-}O}$ (superficial average Ca-O distance). The chemical property included E_{ads} (adsorption energy of As^0), which could reflect CaO's property of capturing As_2O_3.

The Δz_{layers}, $\Delta z_{Ca\text{-}O}$ and E_{ads_mean} was defined as follows:

$$\Delta z_{layers} = | z_{layers\_relaxed} - z_{layers\_fixed} | \quad (1)$$

$$\Delta z_{Ca\text{-}O} = | z_{Ca\text{-}O\_relaxed} - z_{Ca\text{-}O\_fixed} | \quad (2)$$

$$\Delta E_{ads} = | E_{ads} - E_{ads\_mean} | \quad (3)$$

The suffix of 'fixed' represented that the surface layer of slab models was fixed, while 'relaxed' represented the opposite. The suffix of 'mean' represented the mean adsorption energy.

Table S1: Changes in physical and chemical properties of different surface size.

surface size	Δz_{layers}, Å	$\Delta z_{Ca\text{-}O}$, Å	ΔE_{ads}, kJ/mol
1×1	7.9×10^{-2}	7.0×10^{-5}	62.2
2×2	7.9×10^{-2}	6.0×10^{-5}	10.3
3×3	7.8×10^{-2}	6.0×10^{-5}	4.5
4×4	7.7×10^{-2}	6.0×10^{-5}	5.8

The $a \times a$-surface (a=1, 2, 3 and 4) slab models (layer is fixed as 4 layers) were constructed and optimized. The changes in physical and chemical properties are shown in Table 2. Among the models, the 3×3-surface model has the second lowest Δz_{layers} (7.8×10^{-2} Å), the lowest $\Delta z_{Ca\text{-}O}$ (6.0×10^{-5} Å) and the lowest ΔE_{ads} (4.5 kJ/mol). The model surface size was therefore optimized as 3×3. The average adsorption energy E_{ads_mean} did not include the 1×1-surface model due to the obvious large difference (62.2 kJ/mol).

Table S2: Changes in physical and chemical properties of different layers.

layers	Δz_{layers}, Å	$\Delta z_{Ca\text{-}O}$, Å	ΔE_{ads}, kJ/mol
2	7.3×10^{-2}	1.0×10^{-4}	1.1
3	7.1×10^{-2}	9.2×10^{-5}	0.7
4	5.0×10^{-2}	9.0×10^{-5}	0.3
5	7.2×10^{-2}	1.0×10^{-4}	0.9
6	7.3×10^{-2}	1.0×10^{-4}	0.7

The layer thickness was then optimized based on the 3×3-surface size. As shown in Table 3, the 4-layer model has demonstrated acceptable physical and chemistry properties (lowest Δz_{layers}, lowest $\Delta z_{Ca\text{-}O}$, lowest ΔE_{ads}). As the consequent, the 4-layer 3×3-surface slab model was selected to simulate the adsorption of As_2O_3 on CaO surface in this study.

Initial adsorbate structures

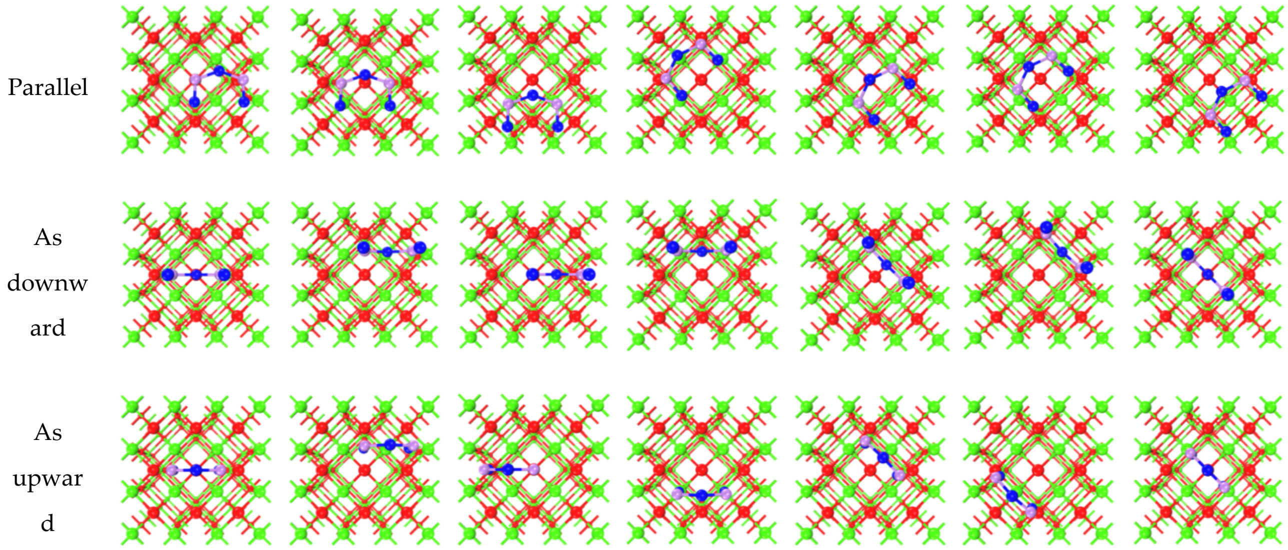

Figure S1: Initial adsorbate structures.

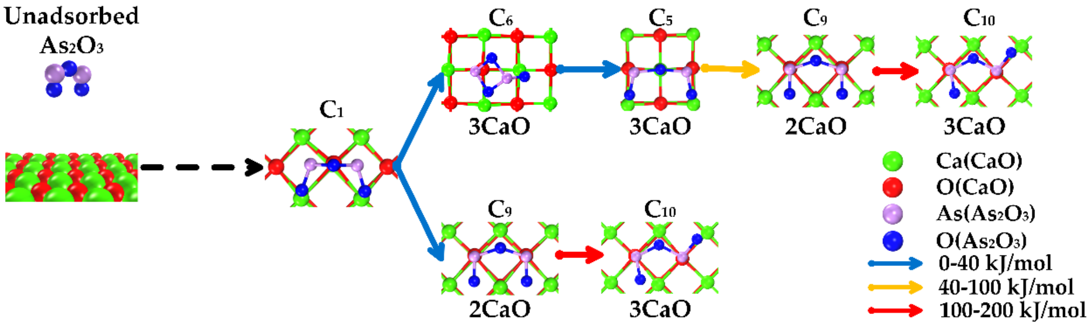

Figure S2: Paths and structures of the physisorption and chemisorption reaction from chemisorption structure 1.

PDOS Analysis

PDOS (Partial Density of States) was calculated to describe the electronic structure changes of As_2O_3 and CaO slab model surface during adsorption. Both the PDOS of physisorption structures and chemisorption structures are selected for discussion here.

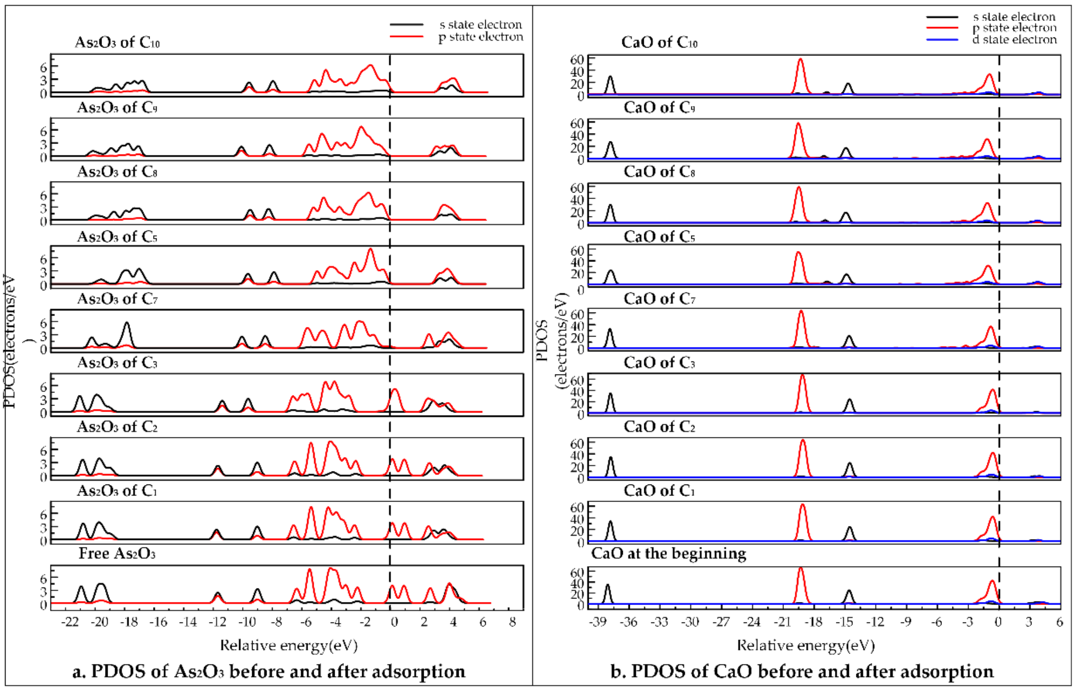

Figure S3: PDOS of As_2O_3 and CaO surface during physisorption and chemisorption (a. PDOS of As_2O_3 molecule; and b. PDOS of CaO surface).

Reference

1. Di Valentin, C.; Pacchioni, G.; Bernasconi, M. Ab initio molecular dynamics simulation of NO reactivity on the CaO (001) surface. *J. Phys. Chem. B* **2006**, *110*, 8357-8362.

2. Xin, G.; Zhao, P.; Zheng, C., Theoretical study of different speciation of mercury adsorption on CaO (001) surface. *Proc. Combust. Inst.* **2009**, *32*, 2693-2699.
